# Toxicity Study and Quantitative Evaluation of Polyethylene Microplastics in ICR Mice

**DOI:** 10.3390/polym14030402

**Published:** 2022-01-20

**Authors:** Sijoon Lee, Kyung-Ku Kang, Soo-Eun Sung, Joo-Hee Choi, Minkyoung Sung, Keum-Yong Seong, Sunjong Lee, Seung Yun Yang, Min-Soo Seo, KilSoo Kim

**Affiliations:** 1 Preclinical Research Center, Daegu-Gyeongbuk Medical Innovation Foundation, Daegu 41061, Korea; sjlee1013@dgmif.re.kr (S.L.); kangkk@dgmif.re.kr (K.-K.K.); sesung@dgmif.re.kr (S.-E.S.); cjh522@dgmif.re.kr (J.-H.C.); tjdalsrud27@naver.com (M.S.); 2 Institute of Animal Medicine & Department of Veterinary Medicine, Gyeongsang National University, Jinju 52828, Korea; 3 Department of Biomaterials Science (BK21 Four Program), Life and Industry Convergence Institute, Pusan National University, Miryang 50463, Korea; ky.seong0124@gmail.com (K.-Y.S.); syang@pusan.ac.kr (S.Y.Y.); 4 Korea Institute of Industrial Technology, Cheonan 31056, Korea; sunjong1774@kitech.re.kr; 5 College of Veterinary Medicine, Kyungpook National University, 80 Daehakro, Buk-gu, Daegu 41566, Korea

**Keywords:** microplastics, polyethylene, toxicity evaluation, quantitative evaluation

## Abstract

The production, use, and waste of plastics increased worldwide, which resulted in environmental pollution and a growing public health problem. In particular, microplastics have the potential to accumulate in humans and mammals through the food chain. However, the toxicity of microplastics is not well understood. In this study, we investigated the toxicity of 10–50 μm polyethylene microplastics following single- and 28-day repeated oral administration (three different doses of microplastics of 500, 1000, and 2000 mg/kg/day) in ICR mice. For the investigation, we administered the microplastics orally for single- and 28-day repeated. Then, the histological and clinical pathology evaluations of the rodents were performed to evaluation of the toxicity test, and Raman spectroscopy was used to directly confirm the presence of polyethylene microplastics. In the single oral dose toxicity experiments, there were no changes in body weight and necropsy of the microplastics-treated group compared with that of controls. However, a histopathological evaluation revealed that inflammation from foreign bodies was evident in the lung tissue from the 28-day repeated oral dose toxicity group. Moreover, polyethylene microplastics were detected in the lung, stomach, duodenum, ileum, and serum by Raman spectroscopy. Our results corroborated the findings of lung inflammation after repeated oral administration of polyethylene microplastics. This study provides evidence of microplastic-induced toxicity following repeated exposure to mice.

## 1. Introduction

The production and use of plastics increased worldwide [1,2]. Approximately 6.6 billion tons of plastic waste was generated globally from 1950 to 2015 [3]. Some of these plastics are recycled but the remainder are dumped into the ocean and landfills, acting as an environmental pollutant. Plastics dumped into the ocean continue to accumulate and spread widely because they are not readily degradable, and hence, are considered an environmental problem. Plastics with a size of less than 5 mm are referred to as microplastics and are classified into primary and secondary microplastics. Primary microplastics are industrially produced and secondary microplastics are small fragments formed when plastics are crushed after exposure to environmental conditions [4]. To date, studies on microplastics mainly focused on whether they are present in the environment, such as in the sea, freshwater, soil, air, and food [5,6,7,8,9,10,11]. Because of their small size and nondegradable, microplastics accumulate inside aquatic organisms. The food chain is the major route through, which humans are exposed to microplastics [12]. In addition, the presence of microplastics in foods, such as sea salt, beer, bottled water, and honey were reported and this represents yet another route through which humans are exposed [13,14]. Microplastics mainly include plastic materials, such as polyethylene, polystyrene, and polypropylene. Polyethylene is the major source of microplastics, and it is present extensively throughout the environment [15,16,17]. Recently, research on microplastics changed from documenting the existence and exposure of microplastics in the environment to whether they have any deleterious effects on organisms in vitro and in vivo. Studies were carried out in aquatic organisms, human-derived cells, and organs of the gut and reproductive system of mammals [18,19,20,21]. According to the size and type of microplastics, there are reports of inflammatory responses, metabolic disorders, cellular damage, and toxicity to specific organs both in vitro and in vivo. Microplastics can accumulate in the gills, liver, and the gastrointestinal tract of aquatic organisms, such as fish and shellfish [22,23]. Studies demonstrated that polystyrene microplastics accumulate in zebrafish and mussels and cause gastrointestinal and liver toxicity [24,25]. Inflammation by polystyrene microplastics was also reported in zebrafish larvae [26]. In addition, there are studies suggesting that microplastics cause reproductive disorders in oysters [27] and neurotoxicity in the zebra mussel, *Dreissena polymorpha* [28]. Polystyrene microplastics induced cytotoxicity and inflammation when they were evaluated using an in vitro culture system of normal human lung epithelial cell lines [29]. In a toxicity study of microplastics using human-derived PBMCs and HMC-1 cells, the expression of inflammatory factors, TNFα, IL-6, and IL-1β, was increased [30]. Repeated oral administration of polystyrene microplastics in mice was shown to induce male reproductive toxicity [31,32] and cause abnormal sperm quality and testicular damage. In addition, repeated administration of microplastics to mice resulted in intestinal damage and caused an imbalance in the intestinal flora [33]. Analysis of microplastics in human feces revealed 9 types of microplastic particles ranging from 50 to 500 μm [34]. These results represent an important clue to the accumulation and release of microplastics following exposure to the human body. Despite these reports, studies on mammals with respect to the toxicological effects of microplastics are limited. In particular, the toxicity test of rodents for evaluating the human risk assessment of the microplastics is presently lacking considering their risk or severity. Therefore, the need and interest in the toxicity evaluation and in vivo accumulation to confirm the in vivo effects of microplastics is increasing. In the present study, we examined the in vivo effects of polyethylene microplastics with a size of 10–50 μm, which is smaller than that detected in human feces. The study was performed by orally administering polyethylene microplastics at single or repeated doses for 28 days and evaluating the toxicity in ICR mice. Histological and clinical pathology evaluations were performed and Raman spectroscopy was used to confirm the presence of polyethylene microplastics in tissues. Our findings provide insight into the in vivo toxicity of polyethylene microplastics and their potential bioaccumulation.

## 2. Materials and Methods

### 2.1. Preparation of Polyethylene Microplastics

Microplastics were prepared from polyethylene beads (5 mm). To prepare 10–50 μm polyethylene particles, we froze polyethylene beads with dry ice (−78 °C). The frozen beads were ground with a homogenizer for 4–5 h. The particles were separated using a 50 μm and a 10 μm mesh filter stepwise and the particles were washed with ethanol 4–5 times. Finally, the particles were dried for 48 h in a 50 °C oven. The average particle size of the polyethylene particles was measured using a Particle Size Analyzer (PSA, ELS-Z2Plus, Otsuka Electronics, Osaka, Japan) and the shape of the microparticles were determined by 3D profile (Confocal microscopy, Keyence, Itasca, IL, USA).

### 2.2. Raman Spectroscopy

Polyethylene microplastics were analyzed using a Raman microscope (RAMANtouch, Nanophoton, Japan) equipped with a laser diode (785 nm). After navigating the morphology of microplastics with a 20× objective lens (Nikon LU Plan Flour 20×/0.45), Raman spectra were collected in the 160–3000 cm^−1^ range using 300 lines per mm grating with 50 μm slit width. The spectrum was measured over a 16-bit dynamic range with Peltier cooled charge-coupled device (CCD) detectors. The acquisition time and number of accumulations were adjusted for each scan to obtain sufficient signals for performing a library search. The spectrometer was calibrated with silicon at a line of 520.7 cm^−1^ prior to spectral gaining. Raw Raman spectra underwent noise reduction by polynomial baseline correction and vector normalization to improve spectral quality (Labspec 6 software, Horiba Scientific). The Raman spectra were compared to that of the SLOPP Library of Microplastics and the spectral library of KnowItAll software (Bio-Rad Laboratories, Inc., Hercules, CA, USA). Similarities above the Hit Quality Index of 80 were considered satisfactory.

### 2.3. Aniumal Treatment and Experimental Conditions

Five-weeks-old male and female ICR mice (68 per gender, KOATECH Inc., Pyeongtaek, Gyunggi-do, Korea) were acclimatized for 1 week. The animals were then divided into groups for a single-dose toxicity study (12 animals per gender, 3 animals per group), a 28-day repeated-dose toxicity study (40 animals per gender, 10 animals per group), and a quantitative evaluation test (16 animals per gender, 4 animals per group). During the experimental period, the animals were acclimated in ventilated IVC cages (395 W × 346 D × 213 H) at a temperature of 22 ± 1 °C, relative humidity of 50 ± 10%, ventilation time of 10–15 h, light for 12 h per day, and an illumination of 150–300 lux. For the single-dose toxicity study, animals in the control group were administered corn oil (Daijung Chemicals Inc, Daejeon, Korea), whereas the animals in the test groups were injected orally with polyethylene microplastics in corn oil at doses of 500, 1000, and 2000 mg/kg (low-, middle-, and high-dose groups) and a dosage of 10 mL/kg. For repeated-dose toxicity studies and quantitative evaluation tests, each group of animals was treated once a day for 28 days in the same manner as the single-dose toxicity study. The two toxicity tests were conducted based on OECD guidelines (408, 423) and the Korea Food and Drug Administration’s Toxic Test Standards Guide (No. 2017-71).

### 2.4. Clinical Observations

Animal observation, the presence of moribund or dead animals, and the measurement of animal weight were conducted once a day, twice a day, and once a week, respectively, for the single-dose and 28-day repeated-dose toxicity studies. Additionally, food and drinking water consumption were measured once a week for the four-week repeated-dose toxicity study.

### 2.5. Necropsy

At the end of the two-week observation period of the single-dose toxicity study, all animals were anesthetized with CO_2_ and exsanguinated through the abdominal aorta. Complete gross postmortem examination was performed on all of the animals. For the 28-day repeated-dose toxicity study, blood from all of the animals was collected from the abdominal aorta under isoflurane (Hana Pharm, Co., Ltd., Seoul, Korea) anesthesia. A complete gross postmortem examination was performed on all animals and tissues (adrenal gland, brain, cecum, colon, duodenum, epididymis, esophagus, heart, ileum, jejunum, kidney, liver, lungs, ovary, pancreas, parathyroid gland, pituitary gland, rectum, spinal cord, spleen, stomach, testis, thymus, thyroid gland, trachea, and uterus) were harvested from male and female mice. After organ extraction, organ weight was measured for the brain, spleen, heart, kidney, liver, testis, epididymis, and ovary. In the quantitative evaluation test, blood was collected from the abdominal aorta under isoflurane anesthesia and tissues (heart, lungs, spleen, liver, kidney, stomach, duodenum, and ileum) were harvested and the organs were weighed.

### 2.6. Clinical Pathology Analysis

Blood samples collected in the 28-day repeated-dose toxicity study were analyzed using a blood cell analyzer (ADVIA 2120i, SIEMENS, Muenchen, Germany) and a serum biochemistry analyzer (TBA 120-FR; Toshiba, JP).

### 2.7. Histopathological Analysis

Tissues harvested from animals from the 28-day repeated-dose toxicity study were fixed in 10% neutral buffered formalin (BBC Biochemicals, Mount Vernon, WA, USA), except for the testis, which were fixed in Davidson’s fixative followed by storage in 10% neutral buffered formalin. For histopathological evaluation, a tissue processor (Thermo Fisher Scientific, Inc., Runcorn, UK) was used to prepare the organs and tissues from the formalin-fixed samples for analysis by fixing, staining, and dehydrating. The paraffin embedded tissue blocks were cut to a 4-μm thickness and mounted onto glass slides. Staining was performed with hematoxylin and eosin using an autostainer (Dako Coverstainer; Agilent, Santa Clara, CA, USA). The histopathological evaluation of all the samples from all animals was conducted in a blind manner.

### 2.8. Quantitative Evaluation of Polyethylene Microplastics in Blood and Tissues

To quantitatively evaluate the number of the polyethylene microplastics in biological samples, the serum and organs were pretreated. After pooling the serum and organ samples, a 10 wt% aqueous KOH solution (20 times the sample weight) was added. The pooled samples were incubated in 37 °C for 48 h with shaking at 250 rpm following homogenization. The samples lysed in KOH solution were filtered stepwise using a stainless-steel filter (47 mm disc, 45 μm pore size) and a silicon filter (1 cm × 1 cm, 1 um pore size) provided by Nanophoton. The number of PE microplastics filtered on the silicon filter was counted using the Raman microscope as described above. Briefly, PE microplastics in the biological samples were scanned by the automated Raman point-by-point mapping mode in both x and y directions on the area of 500 × 375 μm^2^. The number of the total frames per the filtered biological sample was about 534, and the number of PE microplastics per the frame were automatically counted.

### 2.9. Statistical Analysis

All data of the hematology, serum biochemistry, body and organ weight data are presented as the mean ± standard deviation (SD). The statistical significance of the differences between the treated groups and the control group was evaluated by a Student’s *t*-test and one-way analysis of variance using the SAS program (version 9.4 SAS Institute Inc., Cary, NC, USA).

## 3. Results

### 3.1. Characterization of Polyethylene Micrplastics

**Figure 1:**
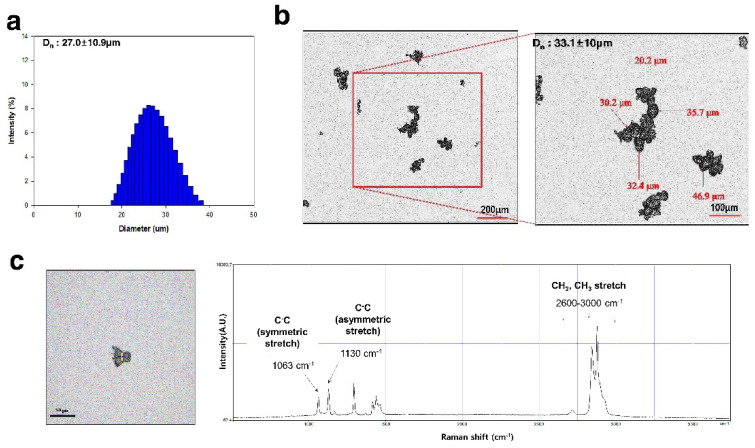
Characteristics of polyethylene microplastics. (**a**) Diameter size of polyethylene microplastics dispersed in ethanol was determined by PSA. (**b**) Shape of polyethylene microplastics were determined by confocal microscopy (Scale bar: 200 and 100 μm). (**c**) Raman spectrum of microparticles filtered on a silicon filter. Scale bar = 50 μm.

According to PSA analysis, the average size of the polyethylene microplastics was 27.0 ± 10.9 μm (Figure 1a). Confocal analysis revealed that the surface of the microplastics was irregular (Figure 1b). After filtering polyethylene dispersed in ethanol, the representative Raman spectrum obtained from filtered microparticles was identified as Polyethylene based on the peaks observed in the region of 1000 cm^−1^ to 1600 cm^−1^, presenting C–C symmetric and asymmetric stretch peaks at 1063 cm^−1^ and 1130 cm^−1^, respectively (Figure 1c). In addition, the methyl CH_2_ groups in Polyethylene is further confirmed by peaks in the region of 2600 cm^−1^ to 3000 cm^−1^ attributed to the CH_2_ and CH_3_ stretching modes [35].

### 3.2. Single Oral Dose Toxicity Study of the Polyethylene Microplastics

**Figure 2:**
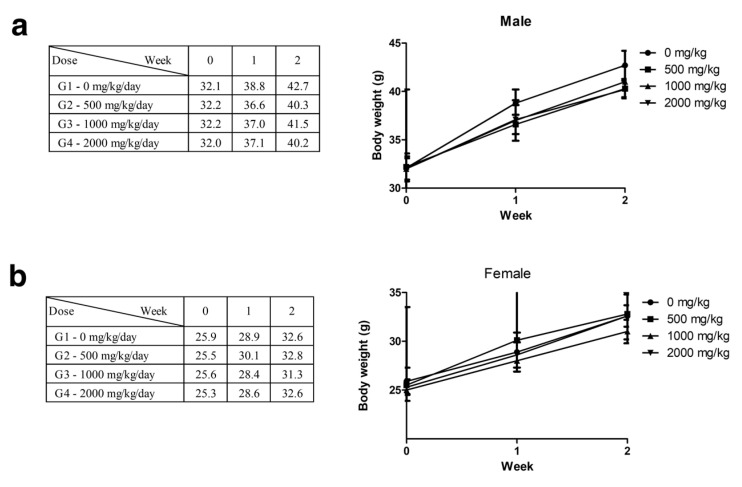
(**a**,**b**) Body weight of male and female mice measured once a week for 2 weeks following a single oral dose of polyethylene microplastics.

During the two-week observation period following a single oral administration of polyethylene microplastics, no specific clinical signs or significant changes in weight were observed in males or females (Figure 2a,b). At the end of the observation period, at necropsy, no changes from the administration of polyethylene microplastics were observed. Therefore, the lethal dose of polyethylene microplastics was determined to be more than 2000 mg/kg.

### 3.3. 28-Day Repeated Oral Dose Toxicity Study of Polyethylene Microplastics

**Figure 3:**
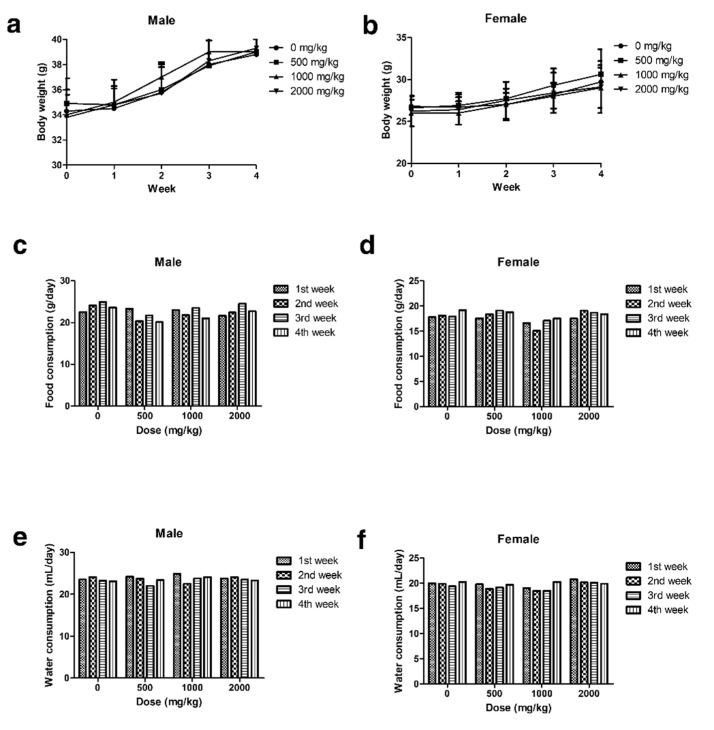
(**a**,**b**) Body weight of male and female mice measured once a week during 4 weeks following a repeated oral dose of polyethylene microplastics. (**c**,**d**) Food and (**e**,**f**) water consumption of male and female mice measured once a week during 4 weeks during a 28-day repeated oral dose toxicity study.

**Figure 4:**
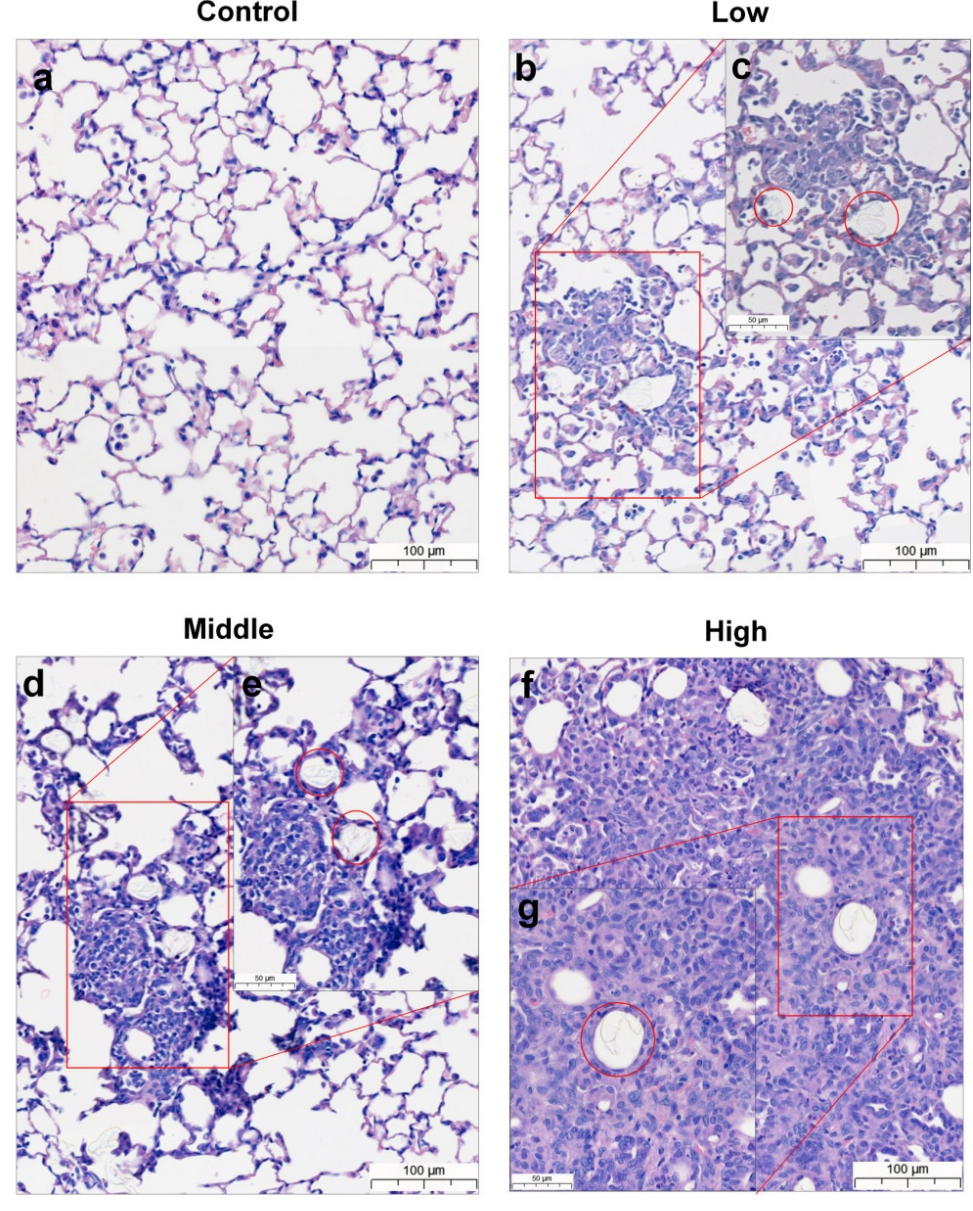
Lung histopathological evaluation of mice from 28-day repeated oral dose toxicity study. Lungs of male mice from (**a**) control group, (**b**,**c**) low-dose group, (**d**,**e**) mid-dose group, and (**f**,**g**) high-dose groups. Left picture represents low magnification, and right is magnified area of red box. Foreign bodies presumed to be polyethylene microplastics in alveolar space of lung (red circle). Scale bar = 100 μm, 50 μm.

**Figure 5:**
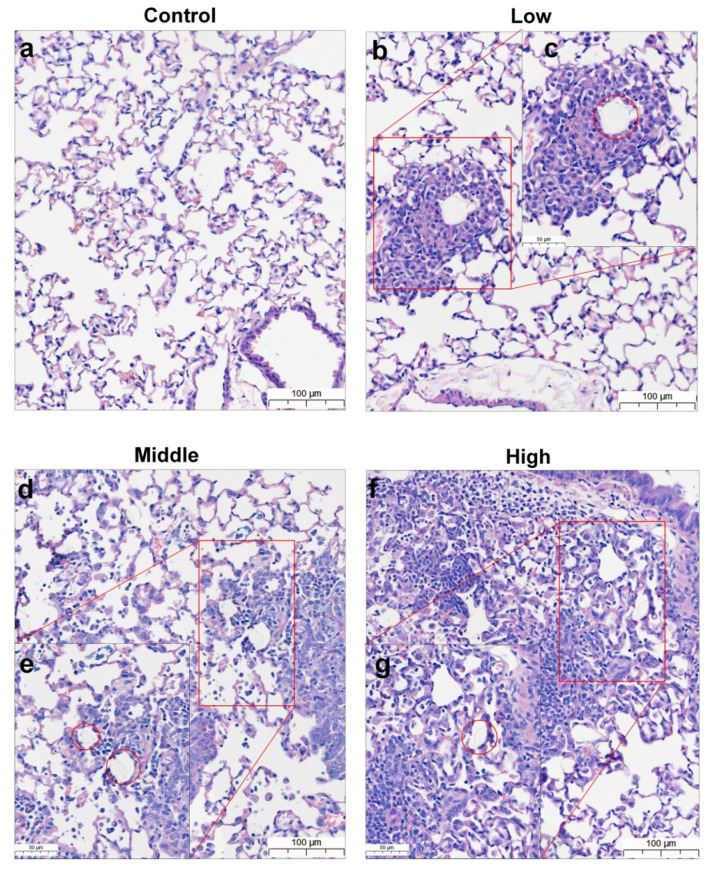
Lung histopathological evaluation of mice from the 28-day repeated oral dose toxicity study. Lungs of female mice from (**a**) control group, (**b**,**c**) low-dose group, (**d**,**e**) mid-dose group, and (**f**,**g**) high-dose groups. Left picture represents low magnification, and right is magnified area of red box. Foreign bodies presumed to be polyethylene microplastics in alveolar space of lung (red circle). Scale bar = 100 μm, 50 μm.

During the observation period of the four-week, repeated oral administration of polyethylene microplastics, no significant changes were observed with respect to clinical signs (Table S1), body weight (Figure 3a,b), food consumption (Figure 3c,d), water consumption (Figure 3e,f), hematological analysis or serum chemistry (Table 1) in male and female mice. Additionally, no changes were observed in absolute or relative organ weight (data not shown). Histopathological evaluation revealed granulomatous inflammation with mixed inflammatory cells (lymphocytes and mononuclear cells) in the alveolar space of the lungs from two females in the low-dose group (500 mg/kg), two males and two females of the middle-dose group (1000 mg/kg), and two males and two females of the high-dose group (2000 mg/kg) (Figure 4b–g and Figure 5b–g, Table 2). Granulomatous inflammation is a cellular response to agents that are difficult to eradicate, such as foreign bodies. These findings in the lungs are thought to represent changes caused by foreign bodies, presumed to be polyethylene microplastics. Therefore, we confirmed that the histopathological findings are caused by the administration of polyethylene microplastics and that an inflammatory reaction occurred because of a toxic reaction to foreign substances. Therefore, in the four-week, repeated oral administration toxicity study of polyethylene microplastics, the no-observed-adverse-effect-level (NOAEL) was estimated to be less than 1000 mg/kg in males and 500 mg/kg in females.

### 3.4. Quantitative Evaluation of Polyethylene Microplastics

**Figure 6:**
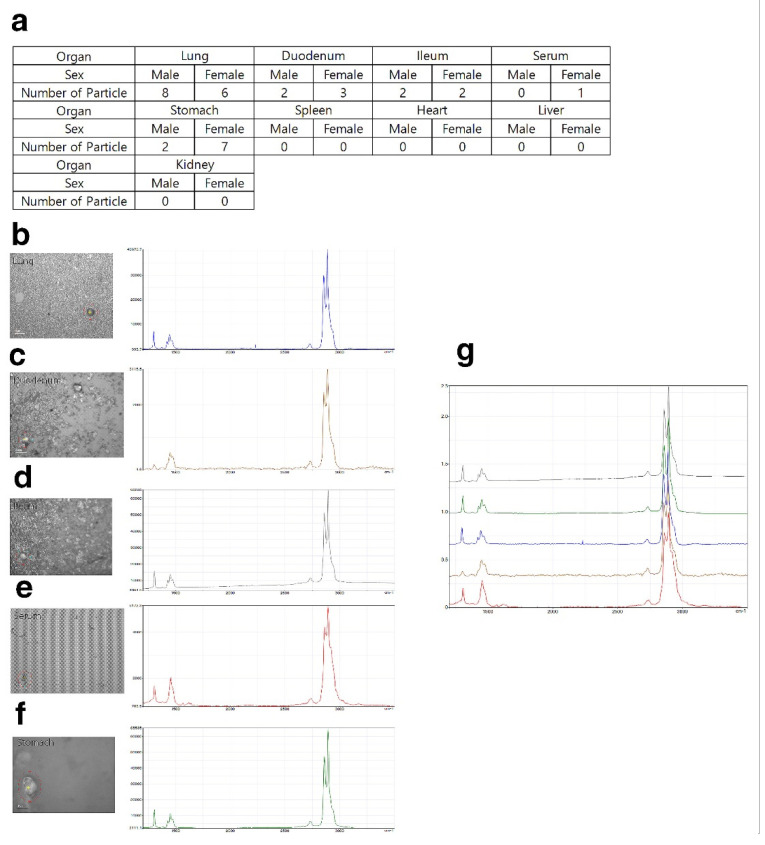
Quantitative evaluation of polyethylene microplastics. (**a**) Number of detected PE microplastic particles in samples from the 2000 mg/kg group. (**b**–**f**) PE microplastics (red circle) detected in lung, duodenum, ileum, serum, and stomach by Raman spectroscopy and wave number of polyethylene microplastics. (**g**) One plot of wave number of (**b**–**f**).

After pretreatment, the harvested organs were analyzed by Raman spectroscopy. No particles were observed in the low- and middle-dose groups (500 and 1000 mg/kg) (data not shown). A total of 14 particles in the lung (8 for males, 6 for females), 1 particle in the serum (1 for female), 9 particles in the stomach (2 for males, 7 for females), 5 particles in the duodenum (2 for males, 3 for females), and 4 particles in the ileum (2 for males, 2 for females) were observed in the high-dose group (2000 mg/kg) (Figure 6a–f). No particles were detected in the liver, spleen, kidney, or heart of the high-dose group.

## 4. Discussion

As the production of plastic products increases, a concomitant increase in plastic waste is inevitable. Plastic waste collects in the ocean and microplastics are formed by weathering and environmental exposure. Microplastics are environmental pollutants and recently attracted significant interest in wider society. In the marine environment, microplastics have the potential to be ingested by aquatic organisms leading to human exposure through the food chain. One study documented the presence and types of microplastics in human feces [36]. Accordingly, there was increased interest in studying the prevalence and effects of environmental microplastics [37,38,39,40,41]. The impact of microplastics was evaluated using aquatic [42], rodent [41] and human [43] cells. Nevertheless, little is known about the toxicity of microplastics. In this study, using standard toxicity evaluation methods (OECD guideline 408, 423), three concentrations of microplastics (500, 1000, and 2000 mg/kg/day) were administered to ICR mice at single and repeated doses for 28 days to evaluate toxicity. In addition, we determined whether the administered microplastics were present in tissues and organs using Raman spectroscopy.

Polyethylene microplastics (PE-MPs) were pulverized to a size of 10–50 μm to increase the similarity with microplastics found in the environment. The fabricated microplastics were atypical and exhibited a fragmented shape (Figure 1). There were many studies using spheroid-shaped microplastics [44,45,46]; however, we considered that microplastics existing in the environment are atypical and have various shapes. Therefore, microplastics that reflect these characteristics were prepared and used in the present study. To confirm the lethal dose50 (LD_50_) of PE-MPs, a single oral dose toxicity study was performed in three groups of mice (500, 1000, and 2000 mg/kg). Clinical signs, body weight, mortality, and gross postmortem evaluation at necropsy showed no significant differences in the treated versus untreated groups (Figure 2). Therefore, as a result of a single oral dose toxicity study of PE-MPs, we established that the LD_50_ was greater than 2000 mg/kg. These data provide insight into the response and effect of mammals to short-term microplastic exposure. Based on the single oral dose toxicity study, a rationale for observing the in vivo effects of repeated administration of microplastics was evident. Three treatment groups (500, 1000 and 2000 mg/kg) were established, and a 28-day repeated oral dose toxicity study was conducted to evaluate the effects of microplastics in mice. No specific changes were observed in the treated groups compared with the control group with respect to body weight, food and water consumption, absolute and relative organ weight, and clinical pathology features. There were no animal deaths in this 28 day, repeated-dose toxicity study (Figure 3, Table 1 and Table 2).

When evaluating toxicity in laboratory animals, spontaneous findings should be distinguished from those caused by the administered substance. For example, prolapse of the penis was observed in one subject of the middle-dose group (500 mg/kg), but it was not a dose-dependent finding. Additionally, no specific findings were observed by necropsy or histopathological analysis of the male reproductive organs compared with the control group. Therefore, this symptom was considered to be a spontaneous finding. According to a previous study [32], polystyrene microplastics can induce male reproductive toxicity. However, we evaluated the toxicity of PE-MPs, thus the applied substance was different compared with that of the previous study [32]. Nonetheless, the evaluation of male reproductive toxicity by each type of microplastic should be the subject of a future study. With respect to wounds on the dorsal skin at high doses in males (Table S1), these findings may result from a conflict among the mice during housing. Individuals with wounds on the dorsal skin were separated into single housing to prevent additional injury [47,48]. According to previous studies, the administration of PE-MPs to mice induces higher anxiety [49]. Therefore, it is important to document that PE-MP exposure can induce behavioral changes. This may provide a clue as to whether there is a correlation between the nervous system and microplastics. Although our study focused on the toxicity of microplastics, it is worthwhile to conduct additional studies on behavioral and neurophysiology.

According to the histopathological results, granulomatous inflammation resulting from foreign bodies in the lungs of the PE-MP-treated group was observed in both males and females (Figure 4 and Figure 5). Inflammation of the lungs is a major finding after repeated administration of PE-MPs for 4 weeks. These results suggest that repeated exposure to PE-MPs causes damage to the lungs. To determine whether the foreign body observed in the lungs was PE-MPs, Raman spectroscopy was performed on the lung tissue and PE-MPs were detected (Figure 6). Using Raman spectroscopy, the shape and number of PE-MPs were confirmed. In addition, PE-MPs were measured in specific regions and revealed that microplastics circulate throughout the body following absorption from the gastrointestinal tract. Raman spectroscopy analysis is a useful method for detecting microplastics and there were reports demonstrating the detection of microplastics in living organisms, the environment, and food using this method [50,51,52,53,54,55,56]. However, to our knowledge, there were no reports that directly demonstrate the detection of microplastics in rodents using Raman spectroscopy, which was done for the first time in our study.

Single and 28-day repeated oral dose toxicity studies were done using PE-MPs. Our study included a clinical pathology and histopathological evaluation and confirmed the presence of PE-MPs in specific tissues. We also established that the lethal dose (LD_50_) was greater than 2000 mg/kg in the single-dose toxicity study using PE-MPs with a particle size of 10–50 μm. The NOAEL value was less than 500 mg/kg in females and 1000 mg/kg in males in the 28-day, repeated oral dose toxicity study. Most importantly, in the case of repeated administration for 28 days, PE-MP was detected in the lungs and inflammation was observed. Raman spectroscopy revealed that PE-MP was present in the lung, gastrointestinal system, and serum. This suggests that microplastics can accumulate in the body and disseminate to specific organs and serum in vertebrates following exposure. This study provides new insights to improve our understanding of the toxicological effects of PE-MP and the biological safety of microplastics to human health following exposure.

To overcome some of the limitations of this study, it will be necessary to evaluate the toxicity of repeated microplastic administration for longer than 28 days. In addition, the study of the toxicological mechanism of microplastics should be done concurrently. Because humans and other organisms are continuously exposed to microplastics through food intake, toxicity evaluations and the effects of long-term exposure to microplastics should be evaluated in future studies.

## 5. Conclusions

We hypothesized that Polyethylene microplastics (PE-MPs) accumulate in the body and cause damage during long-term exposure. The lethal dose of the polyethylene microplastics was determined to be more than 2000 mg/kg through a single dose toxicity study, and no-observed-adverse-effect-level (NOAEL) was less than 1000 mg/kg and 500 mg/kg in male and female mice, respectively. Our results indicate that damage occurred in the lungs when PE-MPs were administered repeatedly for 28 days and microplastics were directly detected in specific tissues and serum from treated mice, which confirms our hypothesis. Further studies will be necessary to identify the molecular mechanisms for the toxicity and effects of long-term exposure to various types of microplastics.

## Figures and Tables

**Table 1:** Clinical pathology analysis in 28-day repeated oral dose toxicity study. Male and female serum biochemistry results and male and female hematology data. Results are expressed as mean ± SD. * *p* < 0.05, ** *p* < 0.01, *** *p* < 0.001 vs. 0.

Group/Dose (mg/kg/day)	Sodium (mmol/L)	Potassium (mmol/L)	Cloride (mmol/L)	Total Protein (g/dL)	Albumin (g/dL)	Blood urea Nitrogen (mg/dL)	Creatinine (mg/dL)	Glucose (mg/dL)	Total Bilirubin (mg/dL)
Sex: Male									
G1 0	155.1 ± 2.2	7.4 ± 2.1	116.8 ± 4.5	4.9 ± 0.3	3.1 ± 0.2	23.7 ± 7.3	0.2 ± 0.1	86 ± 18	0.1 ± 0.1
G2 500	154.4 ± 2.2	7.3 ± 1.6	117.0 ± 1.6	4.9 ± 0.2	3.1 ± 0.1	21.7 ± 3.7	0.2 ± 0.0	84 ± 27	0.1 ± 0.1
G3 1000	155.2 ± 3.1	7.4 ± 2.0	116.8 ± 3.2	5.0 ± 0.2	3.3 ± 0.1	23.9 ± 4.5	0.2 ± 0.0	90 ± 2.6	0.1 ± 0.1
G4 2000	157.5 ± 3.4	7.0 ± 1.3	118.8 ± 3.2	5.0 ± 0.2	3.1 ± 0.3	23.4 ± 4.2	0.2 ± 0.0	101 ± 30	0.1 ± 0.1
Sex: Female									
G1 0	160.1 ± 1.1	7.7 ± 1.5	119.4 ± 2.2	5.2 ± 0.2	3.8 ± 0.1	16.5 ± 3.6	0.2 ± 0.0	63 ± 25	0.0 ± 0.0
G2 500	156.2 ± 2.9 **	7.3 ± 1.1	114.5 ± 3.6 **	4.9 ± 0.3 *	3.6 ± 0.1 *	16.9 ± 2.4	0.2 ± 0.0	67 ± 19	0.0 ± 0.0
G3 1000	154.7 ± 1.6 ***	6.9 ± 1.4	114.3 ± 1.9 ***	5.0 ± 0.3	3.6 ± 0.1	15.3 ± 3.7	0.2 ± 0.0	60 ± 22	0.0 ± 0.0
G4 2000	144.2 ± 1.8	7.6 ± 1.8	108.1 ± 2.2 *	4.8 ± 0.7	3.5 ± 0.5	15.9 ± 3.8	0.2 ± 0.0	81 ± 32	0.0 ± 0.0
**Group/Dose (mg/kg/day)**	**Calcium (mg/dL)**	**Phosphate (mg/dL)**	**Total Cholesterol (mg/dL)**	**Triglycerid (mg/dL)**	**Aspartate Aminotransferase (U/L)**	**Alanin Aminotransperase (U/L)**	**Alkaline Phosphatase (U/L)**		
Sex: Male									
G1 0	9.6 ± 0.3	8.2 ± 1.4	160 ± 24	136 ± 31	65 ± 23	24 ± 6	192 ± 53		
G2 500	9.6 ± 0.2	8.0 ± 0.9	187 ± 48	138 ± 29	61 ± 10	28 ± 10	236 ± 70		
G3 1000	9.5 ± 0.3	8.4 ± 0.8	175 ± 28	147 ± 43	76 ± 27	38 ± 16 *	229 ± 43		
G4 2000	9.7 ± 0.2	8.5 ± 1.3	154 ± 32	135 ± 30	79 ± 21	32 ± 7 *	205 ± 82		
Sex: Female									
G1 0	10.0 ± 0.3	8.4 ± 1.0	108 ± 22	81 ± 26	60 ± 11	18 ± 3	302 ± 75		
G2 500	10.0 ± 0.3 **	7.8 ± 1.2	112 ± 21	93 ± 45	65 ± 30	22 ± 9	274 ± 82		
G3 1000	9.2 ± 0.5 **	7.9 ± 0.8	121 ± 26	93 ± 37	88 ± 52	21 ± 7	278 ± 89		
G4 2000	8.8 ± 1.6 *	7.7 ± 1.4	95 ± 25	79 ± 39	93 ± 52	23 ± 5 *	296 ± 89		
**Group/Dose (mg/kg/day)**	**White Blood Cell (×10^3^ cells/uL)**	**Red Blood Cell (×10^6^ cells/uL)**	**Hemoglobin** **(g/dL)**	**Hematocrit** **(%)**	**Mean Corpuscular Volume** **(fL)**	**Mean Corpuscular Hemoglobin** **(pg)**	**Mean Corpuscular Hemoglobin Concentration** **(g/dL)**	**Red Cell Distribution Width** **(%)**	
Sex: Male									
G1 0	3.77 ± 2.75	8.54 ± 1.75	13.1 ± 4.2	42.3 ± 9.5	49.4 ± 2.3	14.8 ± 3.5	29.9 ± 6.8	13.4 ± 1.3	
G2 500	2.61 ± 0.46	8.93 ± 0.65	13.9 ± 0.8	43.2 ± 2.8	48.4 ± 1.9	15.6 ± 0.7	32.3 ± 0.6	13.1 ± 0.4	
G3 1000	2.55 ± 1.30	9.33 ± 0.32	14.6 ± 0.7	45.0 ± 2.2	48.2 ± 1.3	15.6 ± 0.5	32.4 ± 0.7	12.5 ± 0.5 *	
G4 2000	3.10 ± 1.66	8.96 ± 0.48	14.2 ± 0.7	44.1 ± 2.0	49.3 ± 2.3	15.9 ± 0.9	32.2 ± 0.7	13.0 ± 1.0	
Sex: Female									
G1 0	3.83 ± 2.29	8.78 ± 2.81	14.2 ± 4.5	43.7 ± 14.2	49.5 ± 2.2	16.3 ± 1.0	33.0 ± 1.9	13.6 ± 0.7	
G2 500	2.56 ± 1.16	7.84 ± 3.41	12.4 ± 5.5	39.4 ± 17.2	50.4 ± 1.1	15.9 ± 1.0	31.6 ± 2.2	13.5 ± 0.5	
G3 1000	3.97 ± 1.65	9.35 ± 0.56	13.5 ± 3.9	47.5 ± 3.0	50.4 ± 2.1	14.5 ± 4.4	28.4 ± 8.2	13.4 ± 0.3	
G4 2000	3.36 ± 1.90	7.24 ± 3.97	12.3 ± 5.8	36.7 ± 20.2	50.5 ± 1.6	20.7 ± 16.1	41.3 ± 33.1	13.8 ± 0.9	
**Group/Dose (mg/kg/day)**	**Hemoglobin Distribution Width** **(g/dL)**	**Platelet (×10^3^ cells/uL)**	**Mean platelet volume** **(fL)**	**Neutrophil** **(%)**	**Lymphocyte** **(%)**	**Monocyte** **(%)**	**Eosinophil** **(%)**	**Large Unstained Cell** **(%)**	**Basophil** **(%)**
Sex: Male									
G1 0	2.20 ± 0.15	959 ± 247	4.9 ± 0.3	32.1 ± 12.3	51.0 ± 19.9	2.0 ± 1.5	13.9 ± 16.9	0.8 ± 0.3	0.1 ± 0.1
G2 500	2.40 ± 0.17	1102 ± 317	4.9 ± 0.7	22.5 ± 4.1 *	67.0 ± 7.2 *	1.7 ± 0.7	8.1 ± 7.8	0.7 ± 0.3	0.0 ± 0.1 *
G3 1000	2.25 ± 0.12	1123 ± 223	4.8 ± 0.3	27.3 ± 4.6	56.8 ± 9.1	2.8 ± 1.0	12.4 ± 11.5	0.6 ± 0.2	0.1 ± 0.1
G4 2000	2.19 ± 0.23	1127 ± 192	4.7 ± 0.3	43.6 ± 18.8	45.9 ± 19.7	2.9 ± 0.6	6.7 ± 5.3	0.8 ± 0.4	0.1 ± 0.1
Sex: Female									
G1 0	2.52 ± 0.22	812 ± 380	6.4 ± 2.1	14.4 ± 4.1	71.7 ± 13.5	1.6 ± 0.6	11.3 ± 12.9	0.9 ± 0.4	0.1 ± 0.1
G2 500	2.38 ± 0.10	755 ± 428	6.3 ± 1.4	17.0 ± 2.4	67.5 ± 7.2	1.8 ± 0.7	13.0 ± 8.3	0.6 ± 0.2	0.1 ± 0.1
G3 1000	2.40 ± 0.08	915 ± 196	6.0 ± 0.9	19.2 ± 6.9	60.3 ± 23.3	2.0 ± 0.8	17.5 ± 16.7	0.9 ± 0.3	0.1 ± 0.1
G4 2000	2.63 ± 0.38	751 ± 418	6.9 ± 2.0	16.8 ± 3.2	73.6 ± 5.6	1.8 ± 0.6	7.0 ± 3.3	0.7 ± 0.4	0.1 ± 0.1
**Group/Dose (mg/kg/day)**	**Neutrophil** **(×10^3^ cells/uL)**	**Lymphocyte** **(×10^3^ cells/uL)**	**Monocyte (×10^3^ cells/uL)**	**Eosinophil (×10^3^ cells/uL)**	**Large Unstained Cell** **(×10^3^ cells/uL)**	**Basophil (×10^3^ cells/uL)**	**Reticulocyte (×10^9^ cells/L)**	**Reticulocyte (%)**	
Sex: Male									
G1 0	1.40 ± 1.76	1.86 ± 1.13	0.07 ± 0.05	0.48 ± 0.47	0.03 ± 0.02	0.00 ± 0.00	421.4 ± 144.1	4.88 ± 1.20	
G2 500	0.59 ± 0.13	1.78 ± 0.33	0.05 ± 0.01	0.22 ± 0.24	0.02 ± 0.01	0.00 ± 0.00	356.2 ± 67.5	4.03 ± 0.92	
G3 1000	0.72 ± 0.28	1.52 ± 0.67	0.09 ± 0.06	0.36 ± 0.40	0.02 ± 0.01	0.00 ± 0.00	335.6 ± 52.1	3.60 ± 0.54 **	
G4 2000	1.41 ± 1.02	1.41 ± 1.09	0.09 ± 0.05	0.23 ± 0.25	0.03 ± 0.02	0.00 ± 0.00	296.0 ± 78.8 *	3.31 ± 0.87 **	
Sex: Female									
G1 0	0.51 ± 0.24	2.89 ± 1.96	0.06 ± 0.04	0.34 ± 0.21	0.04 ± 0.04	0.01 ± 0.01	376.3 ± 147.7	4.19 ± 0.78	
G2 500	0.49 ± 0.22	1.97 ± 0.92	0.05 ± 0.02	0.32 ± 0.23	0.02 ± 0.01	0.00 ± 0.00 *	287.2 ± 161.8	3.63 ± 1.09	
G3 1000	0.69 ± 0.24	2.77 ± 1.70	0.07 ± 0.02	0.49 ± 0.25	0.03 ± 0.02	0.00 ± 0.00	364.8 ± 97.7	3.93 ± 1.12	
G4 2000	0.55 ± 0.30	2.47 ± 1.41	0.06 ± 0.05	0.24 ± 0.21	0.03 ± 0.03	0.00 ± 0.00	299.5 ± 199.6	4.00 ± 1.25	

**Table 2:** Histopathology analysis of mice from 28-day repeated oral dose toxicity study. ±: minimal grade; <+>: presence of lesion.

Tissue	Lesion	G1 0 mg/kg		G2 500 mg/kg		G3 1000 mg/kg		G4 2000 mg/kg	
		Male	Female	Male	Female	Male	Female	Male	Female
Epididymis	Sperm granuloma	1 (+)							
Kidney	Vacuolation, tubule				1 (±)				
Liver	Mononuclear cell infiltrate						1 (±)		
Lung including bronchi	Granulomatous inflammation with mixed inflammatory cell infiltration in alveolar space				2 (±)	2 (±)	2 (±)	2 (±)	2 (±)
Pituitary gland	Rathke’s pouch persistant					1 (<+>)			
Stomach	Cyst, glandular stomach							1 (<+>)	

No remarkable findings were observed in other organs (adrenal gland, brain, cecum, colon, duodenum, esophagus, heart, ileum, jejunum, ovary, pancreas, parathyroid gland, rectum, spinal cord, spleen, testis, thymus, thyroid gland, trachea, uterus).

## Data Availability

The data that support the findings of this study are available from the corresponding author upon reasonable request.

## Supplementary Materials

The following supporting information can be downloaded at: https://www.mdpi.com/article/10.3390/polym14030402/s1; Table S1: Clinical signs associated with the 28-day repeated oral dose toxicity study.
