# Supplementary material for: Toxicity Study and Quantitative Evaluation of Polyethylene Microplastics in ICR Mice
Source: Polymers (Basel). 2022 Jan 20;14(3):402. doi: 10.3390/polym14030402 (PMC8839995; doi:10.3390/polym14030402)
Supplement: Supplementary file 1 [file polymers-14-00402-s001.zip › polymers-1543589-supplementary.pdf]

Table S1. Clinical signs associated with the 28-day repeated oral dose toxicity study.

a

Sex: Male

Group / Dose (mg/kg/day)	No. of animals	Clinical sign	Day																											
			1	2	3	4	5	6	7	8	9	10	11	12	13	14	15	16	17	18	19	20	21	22	23	24	25	26	27	28
G1<br>0	10	NOA	10	10	10	10	10	10	10	10	10	10	10	10	10	10	10	10	10	10	10	10	10	10	10	10	10	10	10	10
G2<br>500	10	NOA	10	10	10	10	10	10	10	10	10	10	10	10	10	10	10	10	10	10	10	10	10	10	10	10	10	10	10	10
G3	10	NOA	10	10	10	10	10	9	9	9	9	9	9	9	9	9	9	9	9	9	9	9	9	9	9	9	9	9	9	9
1000		prolapse of penis						1	1	1	1	1	1	1	1	1	1	1	1	1	1	1	1	1	1	1	1	1	1	1
G4	10	NOA	10	10	10	10	10	7	7	7	5	5	5	5	5	5	5	5	5	5	5	5	5	5	5	5	5	5	5	5
2000		wound, back						3	3		2	2																		
		crust, back								3	3	3	5	5	5	5	5	5	5	5	5	5	5	5	5	5	5	5	5	5

Sex: Female

Group / Dose (mg/kg/day)	No. of animals	Clinical sign								Day														Day						
			1	2	3	4	5	6	7	8	9	10	11	12	13	14	15	16	17	18	19	20	21	22	23	24	25	26	27	28
G1<br>0	10	NOA	10	10	10	10	10	10	10	10	10	10	10	10	10	10	10	10	10	10	10	10	10	10	10	10	10	10	10	10
G2<br>500	10	NOA	10	10	10	10	10	10	10	10	10	10	10	10	10	10	10	10	10	10	10	10	10	10	10	10	10	10	10	10
G3<br>1000	10	NOA	10	10	10	10	10	10	10	10	10	10	10	10	10	10	10	10	10	10	10	10	10	10	10	10	10	10	10	10
G4<br>2000	10	NOA	10	10	10	10	10	10	10	10	10	10	10	10	10	10	10	10	10	10	10	10	10	10	10	10	10	10	10	10
